# Rapamycin Regulates iTreg Function through CD39 and Runx1 Pathways

**DOI:** 10.1155/2014/989434

**Published:** 2014-03-31

**Authors:** Yunjie Lu, Jirong Wang, Jian Gu, Hao Lu, Xiangcheng Li, Xiaofeng Qian, Xiaoshan Liu, Xuehao Wang, Feng Zhang, Ling Lu

**Affiliations:** Translational Medicine Research Center of Jiangning Hospital and Liver Transplantation Center of First Affiliated Hospital, Nanjing Medical University, Nanjing 210029, China

## Abstract

It has been shown that rapamycin is able to significantly increase the expression of FoxP3 and suppress activity in induced Treg (iTreg) cells *in vivo* and *in vitro*. CD39 is a newly determined Treg marker that relates to cell suppression. Runx1, a regulator of FoxP3, controls the expression of adenosine deaminase (ADA) gene, which is found recently in the downstream of CD39 pathway in trophoblast cells. Whether rapamycin would influence CD39 pathway and regulate the expression of Runx1 remains to be determined. The addition of rapamycin to human CD4^+^ naïve cells in the presence of IL-2, TGF-**β** promotes the expression of FoxP3. In this paper, we found that CD39 positively correlated with the FoxP3 expression in iTreg cells. Rapamycin induced iTreg cells showed a stronger CD39/Runx1 expression with the enhanced suppressive function. These data suggested that CD39 expression was involved in iTreg generation and the enhanced suppressive ability of rapamycin induced Treg was partly due to Runx1 pathway. We conclude that rapamycin favors CD39/Runx1 expression in human iTreg and provides a novel insight into the mechanisms of iTreg generation enhanced by rapamycin.

## 1. Introduction

CD4^+^CD25^+^FoxP3^+^ regulatory T cells (Treg) play an essential role in maintaining immunological homeostasis. Forkhead box P3 transcription factor (FoxP3) is responsible for the differentiation and function of Treg cells [1]. CD4^+^FoxP3^+^ T cells show a greater suppressive ability and immune function than normal T cells* in vivo *and* in vitro*. CD4^+^Foxp3^+^ T cells can be divided into two subsets, thymus-derived natural regulatory T cells (nTreg) and induced Treg cells (iTreg). In clinical trials, the major limitation of nTreg is the poor population of peripheral circulating CD4^+^ T cells, while iTreg shows a good alternative to nTreg with a greater proliferative ability [2]. iTreg can be induced from naïve CD4^+^CD25^−^ T cells in both mice and human. Recently reports have shown that iTreg plays an important role in treating various autoimmune diseases in mice including autoimmune diabetes, experimental arthritis, and other immune-mediated inflammatory diseases [3, 4].

CD39 is an ectoenzyme that hydrolyzes ATP and adenosine diphosphate (ADP) into adenosine monophosphate (AMP) and is localized on the surface of endothelial cells and circulating platelets. Now it is determined as an activation marker of lymphoid cells and expressed in human CD25^+^FoxP3^+^ Treg cells [5]. CD39 has also been elucidated to be related to suppressive function of Treg [6, 7]. Researches show that the expansion of CD39^+^ Treg inhibits IL-2 expression in activated T cells and correlates directly with immune activation in AIDS patients [8, 9]. Adenosine deaminase (ADA) which is able to catabolize adenosine plays an obbligato role in CD39-CD73-adenosine pathway [10]. ADA deficiency may result in a fatal severe combined immunodeficiency disease (SCID) [11].

Runt-related transcription factor 1 (Runx1) belongs to a small family of transcription factors, including Runx1, Runx2, and Runx3, and is composed of an NH2-terminal DNA-binding runt homology domain followed by a transcriptional activation domain and COOH-terminal negative regulatory domain [12, 13]. The study with respect to Runx1 has focused largely on its indispensable effect on FoxP3 expression and Treg function [14]. Knockdown of Runx1 by siRNA eliminates the ability of Treg cells to suppress T effector cells* in vitro* [13]. A previous study demonstrates that the Runx1 transcription factor plays a significant role in regulating ADA gene expression in the trophoblast cells [15].

Rapamycin has been demonstrated to be an immune modulator which prevents graft rejection in transplant patients [16, 17]. Previous studies have shown that rapamycin improves the FoxP3 expression and selectively expands the functional Treg cells both* in vivo *and* in vitro *with the appropriate suppressive activity [18–21]. However, it is not clear that how rapamycin influences CD39 and Runx1 pathways in human iTreg cells.

## 2. Materials and Methods

### 2.1. Isolation of Naïve T Cells

Peripheral blood mononuclear cells (PBMCs) were prepared from heparinized venous blood of healthy adult volunteers by Ficoll-Hypaque density gradient centrifugation. All protocols that were involved in human blood donors were approved by Nanjing Medical University. CD4^+^CD45RA^+^ naïve T cells were isolated from PBMC with human naive CD4^+^ T-Cell Isolation Kit II (Miltenyi Biotec) by MACS. The purity of selected cells was routinely more than 95% as determined by flow cytometry.

### 2.2. Generation of Human iTreg Cells* In Vitro*

Fresh naïve T cells from PBMC were stimulated with anti-CD3/CD28 beads (Life Technologies) at a bead: T-cell ratio of 3 : 1 in the presence of IL-2 (100 U/mL), TGF-*β* (10 ng/mL), and rapamycin (100 ng/mL) in different conditions. The concentration of naïve T cells was 0.5 million/mL at the beginning. All the cells were incubated at 37°C for 7 days. IL-2 (100 U/mL) was renewed every 2 days.

### 2.3. Flow Cytometric Assays

All the cells were analyzed by flow cytometry after staining with the following antibodies (all from BD-Biosciences): anti-human CD4, CD25, CD28, CD39, and CD127. For FoxP3 staining, cells were first stained with surface antibodies, then fixed/permeabilized in cytofix/permeabilization solution (Biolegend), and stained with anti-human FoxP3.

### 2.4. Real-Time PCR

Total RNA was extracted with RNA simple total RNA kit (Tiangen Biotech), and cDNA was obtained using RT-Master Mix (TaKaRa). mRNA levels were quantified with SYBR Premix Ex Taq (TaKaRa). Primer sequences were as follows (18S as internal control):

 ADA, 5′-TTCCTTCCAAGAAGACCATGA-3′ and
 5′-GGTTTCAGATTCAACCATGC-3′;
 Runx1, 5′-GGACGCCAGAAGGAAGTCAA-3′ and
 5′-TCGGACCACAGAGCACTTTC-3′;
 18S, 5′-CTCTTAGCTGAGTGTCCCGC-3′and
 5′-CTGATCGTCTTCGAACCTCC-3′.

### 2.5. Suppressive Assays of CD4^+^ Treg Cells* In Vitro*

PBMC was isolated as described previously and labeled with CFSE (Invitrogen). Anti-CD3 mAb-coated beads (Dynal) were added at 1 : 1 (bead : PBMC), and washed iTreg cells were added at ratios from 1 : 2 to 1 : 32 (Treg : PBMC). Finally cultures were incubated at 37°C. On day 4, cells were stained with anti-CD8 APC. Data was acquired and analyzed using the proliferation platform in FlowJo, and suppression index was determined using division index.

### 2.6. Statistical Analysis

Statistical analysis was performed using GraphPad Prism 5.0 software. Data was presented as mean ± SEM. Evaluation of differences between two groups was evaluated using Student's *t*-test. *P* < 0.05 was considered as statistically significant difference.

## 3. Results

### 3.1. CD39 Expression in CD4^+^ T Cells in Human Peripheral Blood

**Figure 1:**
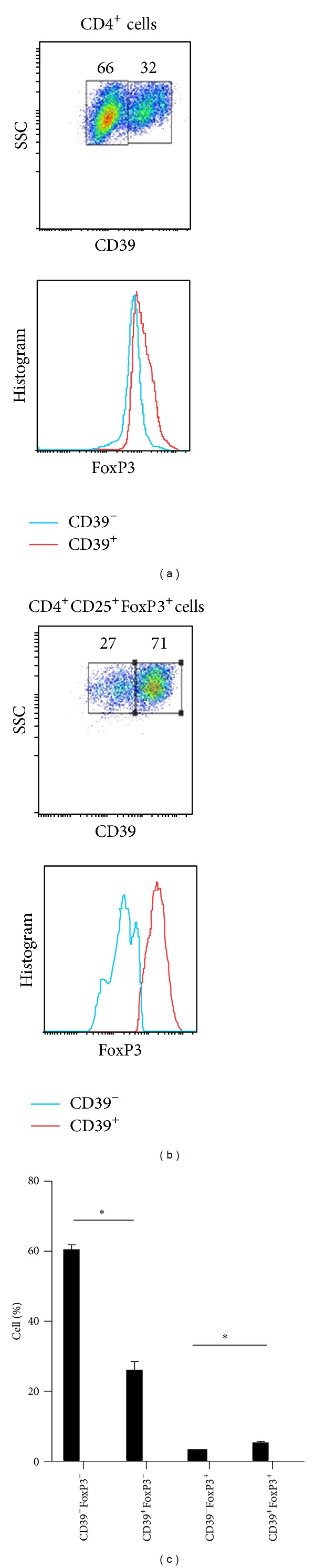
CD39 expression in human peripheral blood and nature FoxP3 cells. (a) Representative expression of CD39 gated in CD4^+^ T cells. (b) Representative expression of CD39 gated in CD4^+^CD25^+^FoxP3^+^ cells. (c) Different proportions of CD39^+^ and FoxP3^+^ cells in CD4^+^ T cells. The values indicated the mean ± SEM of 3 separate experiments. *P < 0.05.

A recent report shows that CD39 is related to T-cell proliferation and FoxP3 function [22]. However, whether CD39 plays an important role in maintaining human immunologic homeostasis is still unknown. To answer this question, initially, we evaluated the frequencies of CD39 in the human CD4^+^ T cells and Treg cells. Peripheral blood mononuclear cells (PBMCs) were isolated from 3 healthy donors, and the expression of CD4, CD25, CD39, and FoxP3 was analyzed by flow cytometry. Approximately 30% of CD4^+^ T cells were CD39 positive and these CD4^+^CD39^+^ T cells revealed an enhanced FoxP3 expression compared to CD4^+^CD39^−^ T cells (Figure 1(a)). Next, we gated on the CD4^+^CD25^+^FoxP3^+^ Treg cells to calculate the CD39 expression in CD4^+^FoxP3^+^ T cells. As depicted in Figure 1(b), more than 70% of Treg cells were CD39 positive. Furthermore, FoxP3 expression was significantly distinct in CD39^+^ Treg cells compared to CD39^−^ cells.  Figure 1(c) demonstrated the different proportion of the cells mentioned above. Here we conclude that CD39 is highly expressed in human FoxP3^+^ T cells and positively correlates with the FoxP3 expression in human peripheral blood.

### 3.2. The Addition of Rapamycin Improves the Expression of FoxP3 and Develops the Potent Suppressive Activity* In Vitro*

**Figure 2:**
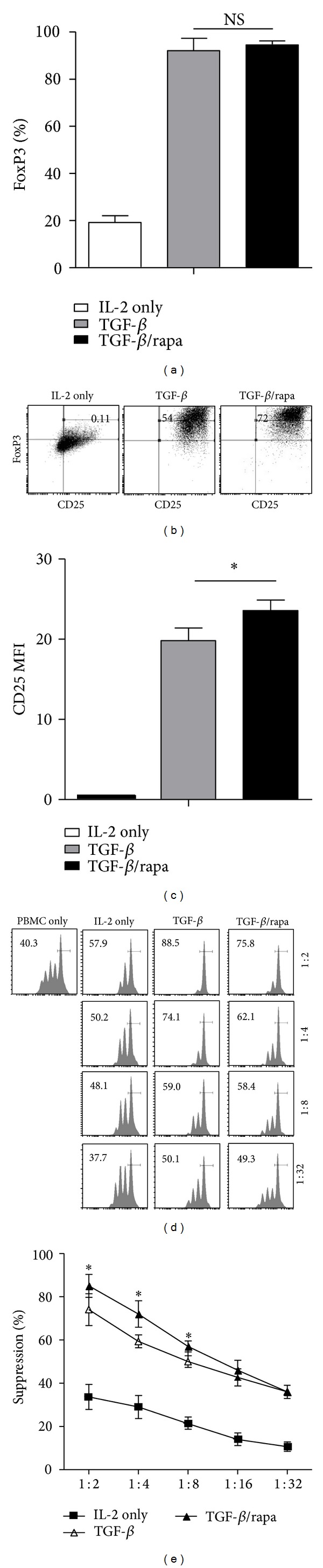
Rapamycin improved the expression of FoxP3 and developed the potent suppressive activity* in vitro*. (a) The proportion of CD4^+^CD25^+^FoxP3^+^ iTreg induced from naïve T cells. (b) CD25 and FoxP3 coexpression in iTreg was assessed by flow cytometry on day 7. (c) Relative CD25 MFI in iTreg cells on day 7 with or without rapamycin. (d) In this representative experiment, the cells were stained for anti-CD8 and the suppressive activity of various primed CD4^+^ cells subsets on CFSE-labeled CD8^+^ at various T suppressor to T effector ratios was shown. (e) The mean ± SEM percent suppression of iTreg at the various ratios. The values indicated the mean ± SEM of 3 separate experiments. *P < 0.05.

Naïve CD4^+^ T cells were cultured with suboptimal anti-CD3/CD28 beads in the presence of IL-2, with or without TGF-*β* and rapamycin for 7 days. While IL-2 and TGF-*β* increased the percentage of CD4^+^CD25^+^FoxP3^+^ T cells, the addition of rapamycin markedly enhanced this effect (Figures 2(a) and 2(b)). These data were consistent with previous studies, which showed that rapamycin favored Foxp3 expression and promoted the suppressive activity of iTreg cells [19–21, 23]. Then, we observed a distinct population of FoxP3^high^ cells, which is defined as FoxP3^++^ cells. The percentage of FoxP3^++^ iTreg cells was obviously increased from 13.6% to 27.8% in the presence of rapamycin (Figure 2(b)). The expression of CD25 was shown as the mean fluorescence intensity (MFI) for each culture condition which showed that rapamycin also enhanced CD25 expression* in vitro* (Figure 2(c)). Since the ability of cell suppression was important for the treating effect of iTreg cells, CFSE coculture assays were performed to estimate the suppressive ability of rapamycin-expanded Treg cells. Washed iTreg cells were coincubated with CFSE-labeled fresh PBMC in the presence of anti-CD3 beads. Although there was no significant difference between the iTreg cultured with or without rapamycin in low ratio (1 : 32), rapamycin did improve suppressive activity in high ratio (from 1 : 2 to 1 : 8) compared to IL-2 and TGF-*β* group (Figures 2(d) and 2(e)). On the whole, rapamycin improves the FoxP3 expression and enhances suppressive activity* in vitro*.

### 3.3. Rapamycin Improves CD39 Expression in iTreg Cells

**Figure 3:**
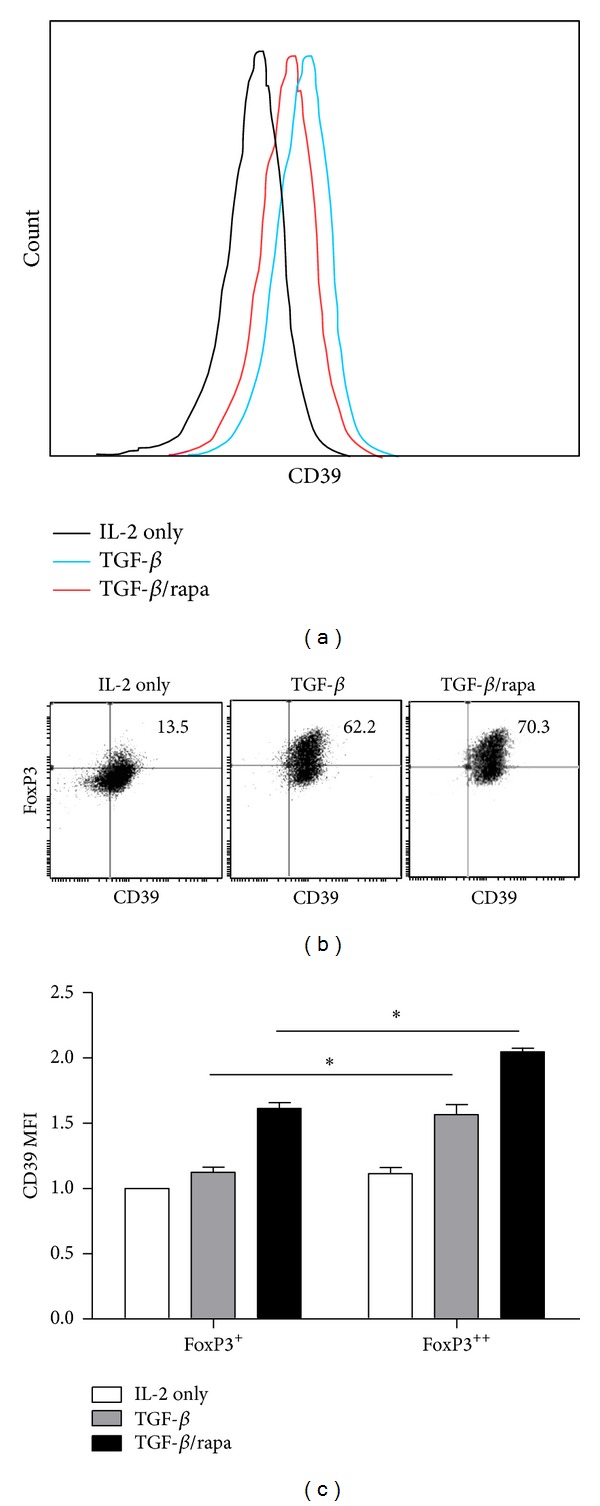
Rapamycin improved CD39 expression in iTreg cells. (a) Representative intensity of CD39 in iTreg cells. (b) FACS analysis of CD39 and FoxP3 expression with or without rapamycin was shown. (c) Relative CD39 MFI in FoxP3^+^ and FoxP3^++^ iTreg cells on day 7 with or without rapamycin. The values indicated the mean ± SEM of 3 separate experiments. *P < 0.05.

As CD39 is important for the function of iTreg [24], next we evaluated the expression of CD39 in iTreg cultured in different conditions. TGF-*β* enhanced the expression of CD39 from 14% to 62% compared with the group with IL-2 alone, while the proportion ascended to nearly 70% with the addition of rapamycin (Figures 3(a) and 3(b)). CD39 MFI (Figure 3(c)) were also detected in these Treg cells which confirmed that the expression of CD39 and FoxP3 was positively correlated and enhanced by rapamycin. Therefore, we suggest that rapamycin improves CD39 expression in iTreg cells.

### 3.4. Rapamycin Upregulates ADA and Runx1 mRNA Level in iTreg Cells

**Figure 4:**
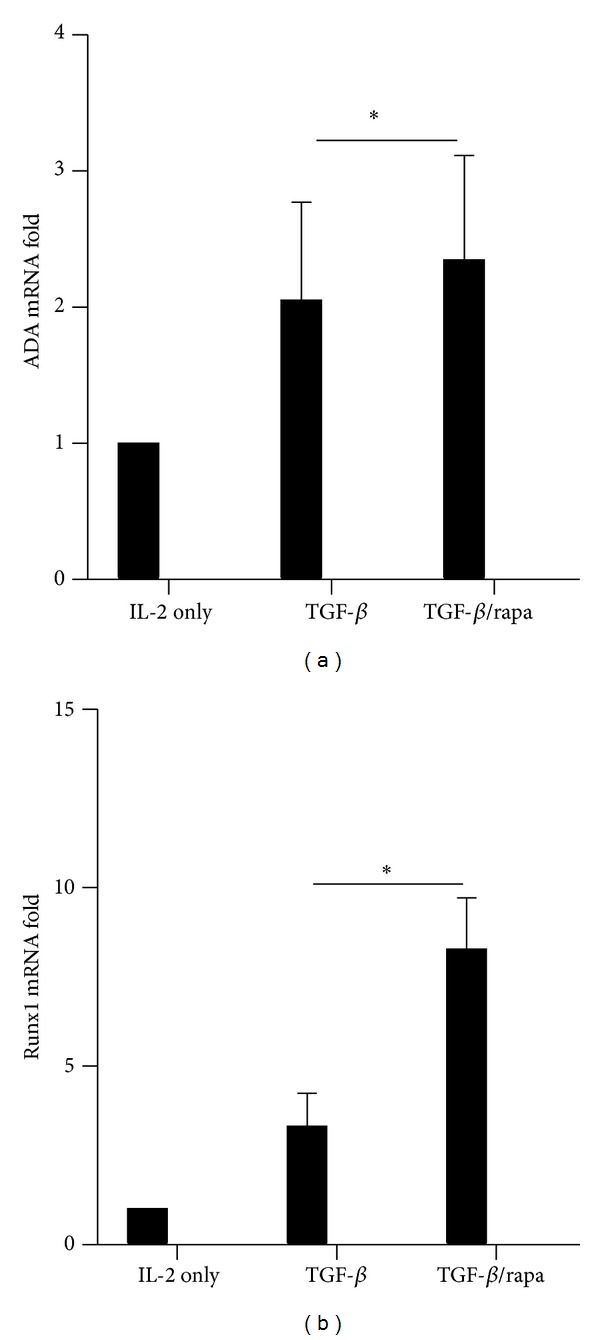
Rapamycin upregulated the mRNA level of ADA and Runx1 in Treg cells. The expression of (a) ADA mRNA and (b) Runx1 mRNA was determined by RT-PCR. Gene expression levels from the IL-2 group were set at 1. The values indicated the mean ± SEM of 3 separate experiments. *P < 0.05.

ADA and Runx1 play essential roles in FoxP3 expression and Treg function [13, 14, 25]. A previous study reveals that Runx1 regulates ADA gene expression in the trophoblast cell line [15]. RT-PCR was performed to estimate the mRNA level of ADA; Figure 4(a) showed that the mRNA level of ADA was almost doubled in TGF-*β* group compared to IL-2 group and rapamycin slightly increased the ADA mRNA level. Next, we detected Runx1 expression in the mRNA level, Figure 4(b) demonstrated that Runx1 expression was increased approximately 3-fold by TGF-*β*, and rapamycin obviously increased Runx1 mRNA level. Since FoxP3 expression is controlled by Runx1 [14], here we found that Runx1 expression was significantly enhanced by rapamycin. This observation suggests that rapamycin upregulates ADA and Runx1 in human iTreg cell induction.

### 3.5. Phenotypic Characterization of Human CD39^+^ iTreg Cells

**Figure 5:**
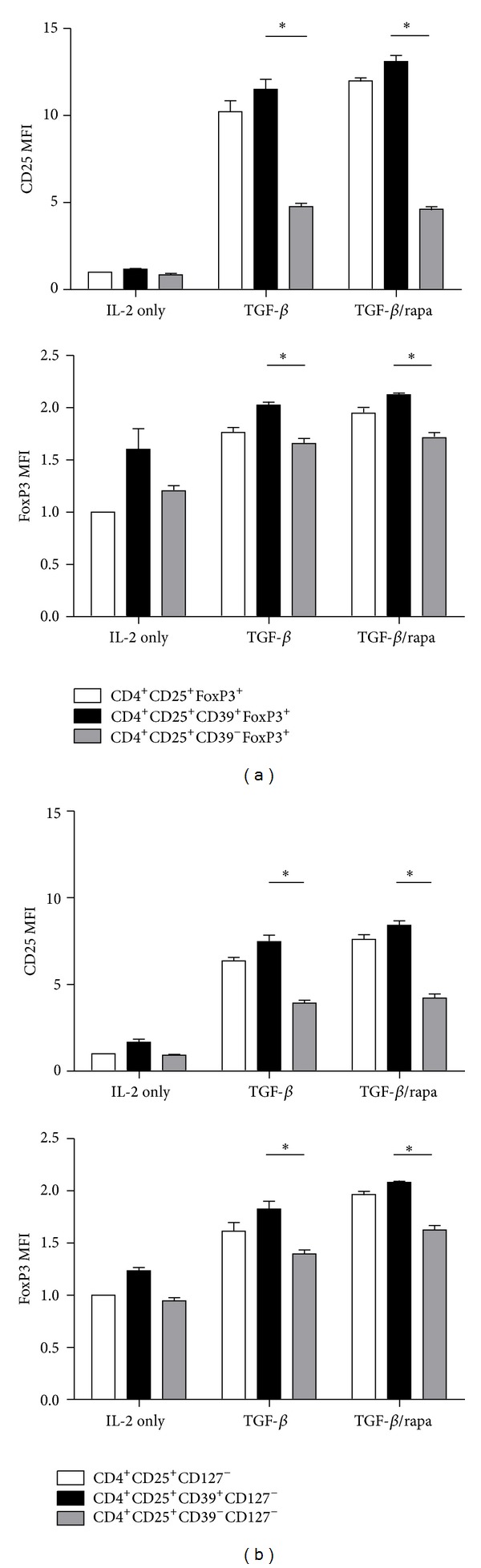
Rapamycin induced CD39^+^ iTreg cells showed a greater CD25 and FoxP3 expression. Relative CD25 and FoxP3 MFI of CD25^+^CD39^+^FoxP3^+^ and CD25^+^CD39^−^FoxP3^+^ in (a) CD4^+^CD25^+^FoxP3^+^ iTreg cells and (b) CD4^+^CD25^+^CD127^−^ iTreg cells. The values indicated the mean ± SEM of 3 separate experiments. *P < 0.05.

We elucidated that rapamycin promotes CD39 expression and then assumed that CD39^+^ iTreg cells might be a new subset of iTreg cells. To see phenotypic characterization of human CD39^+^ iTreg cells, we calculated MFI for CD25, FoxP3 in different Treg phenotypes. As shown in Figure 5(a), a stronger CD25 and FoxP3 expression was detected in CD39^+^ iTreg cells compared to CD39^−^ iTreg in the same culture condition. In rapamycin group, the relative FoxP3 MFI in CD39^+^ iTreg was about 20% higher than CD39^−^ iTreg. Since CD127 could be an effective surface marker for CD4^+^CD25^+^FoxP3^+^ Treg cells in flow sorting, we also determined the expression of CD39 in CD4^+^CD25^+^CD127^−^ iTreg cells. CD4^+^CD25^+^CD39^+^CD127^−^ iTreg also showed a significant difference of FoxP3 and CD25 expression compared to CD4^+^CD25^+^CD39^−^CD127^−^ iTreg cells (Figure 5(b)), and the relative FoxP3 MFI in CD4^+^CD25^+^CD39^+^CD127^−^ iTreg was also about 20% higher than CD4^+^CD25^+^CD39^−^CD127^−^ iTreg. Taken together, we demonstrate that CD4^+^CD25^+^CD39^+^CD127^−^ iTreg cells show a stronger FoxP3 expression and CD39 could be an additional marker for Treg cell sorting.

## 4. Discussion

CD39 is a newly determined Treg marker that relates to cell suppression [26]. CD39^+^ Treg subset mediates a higher suppression compared to control HIV patients [8, 27]. However, CD39 is also expressed in activated T cells [28]. Herein we proved that more than 30% of CD4^+^ T cells in human PBMC were CD39 positive, while CD4^+^CD39^−^ iTreg cells showed a low frequency of FoxP3 compared to CD4^+^CD39^+^ iTreg. Thus, we demonstrate that CD39 is involved in FoxP3 expression and Treg cells in human CD4^+^ T cells.

ATP inhibits the generation and function of regulatory T cells [29]. CD39 which plays a crucial role in immunological system by generating adenosine and removing ATP becomes a promising therapeutic target in oncology [5]. ADA is involved in this pathway and converts adenosine and deoxyadenosine into inosine and deoxyinosine. CD4^+^CD25^high^ T cells express low level of ADA compared with effect T cells [30]. Adenosine and deoxyadenosine would accumulate in cells and then lead to ADA-SCID in the absence of ADA [25]. Thus, it might need a balanced metabolism of adenosine in Tregs because both overexpression and underexpression of ADA would cause Treg function unbalance. We demonstrate that rapamycin would obviously increase CD39 expression in iTreg cells and also enhance FoxP3 expression and suppressive function. A slightly enhanced ADA mRNA level was also observed in our study, which might be a positive feedback along with the increased CD39 expression. CD39/adenosine pathway is important to the balance of activation and regulation of effect immune responses. Since we found that CD39^+^CD127^−^ iTreg cells acquired a stronger FoxP3 expression compared to CD39^−^CD127^−^ iTreg cells, it provides us with a new marker for novel strategy of flow cell sorting.

Rapamycin is an inhibitor of mTOR pathway, which is able to favor the proliferation of Treg cells [31]. Here we conclude that iTreg induced from naïve T cells would acquire an enhanced CD25 and FoxP3 expression in the presence of rapamycin.

Rapamycin promotes the demethylation of Treg cells in the TSDR region and improves FoxP3 expression and suppressive activity [32]. Runx1 is also proved to be an unreplaceable gene which controls FoxP3 expression and Treg function [13, 33]. Recently, Strober W's group discovered that ROR*γ*t which is related to TH17 cells can also be induced by Runx1 [34]. Thus, Runx1 may have a complicated mechanism in balancing the generation of FoxP3 and IL-17 in human naïve cells. We found that the number of FoxP3^++^ Treg was greater in rapamycin group and Runx1 expression was upregulated by rapamycin. It is consistent with the conclusion that rapamycin promotes FoxP3 expression and restrain IL-17 by altering expression of ROR*γ*t [35]. However, more researches are required to reveal the mechanism that how Runx1 regulates Treg and Th17 cells.

Human Treg shows a great suppressive effect and stability in treating autoimmune disease. However, because of the limited amount of these cells in the blood, there are technical difficulties to expand their clinical usage. The well-described protective effect of CD39^+^ iTreg induced effectively by rapamycin provides us with a new strategy for Treg generation. Rapamycin greatly enhanced the expression of CD39 and Runx1. ADA, a promoter of CD39 pathway, can also be activated by rapamycin. Rapamycin treated CD39^+^ iTreg may be an alternative choice which can be used for autoimmune disease treatment* in vivo*. Finally, we conclude that CD39 as a Treg function marker could provide us with new insights into clinical cell therapy in autoimmune diseases.

## 5. Conclusion

Our study indicated that the CD39 and Runx1 signaling pathways were involved in the induction of human iTreg by rapamycin. Rapamycin enhanced the FoxP3 expression and suppressive activity of iTreg with the elevated ADA and Runx1 expression; CD39 correlated positively with FoxP3 expression which proved to be a promising marker for human Treg cell sorting.
